# Kaempferol activates chloride secretion via the cAMP/PKA signaling pathway and expression of CFTR in T84 cells

**DOI:** 10.3389/fphar.2024.1401273

**Published:** 2024-09-11

**Authors:** Janjira Thaweewattanodom, Chatsri Deachapunya, Sutthasinee Poonyachoti

**Affiliations:** ^1^ Department of Physiology, Faculty of Medicine, Srinakharinwirot University, Bangkok, Thailand; ^2^ Department of Physiology and CU-Animal Fertility Research Unit, Faculty of Veterinary Science, Chulalongkorn University, Bangkok, Thailand

**Keywords:** kaempferol, T84 cells, Cl^−^ secretion, short-circuit current, apical Cl^−^ current

## Abstract

Kaempferol is a flavonol identified as the most potent activator of chloride (Cl^−^) secretion among other flavonoids in airway epithelial cells. This study aimed to investigate the cellular mechanisms by which kaempferol stimulates Cl^−^ secretion in the T84 human colon carcinoma cell line by Ussing chambers and voltage clamp technique. Bilateral addition of kaempferol (1–100 µM) increased short-circuit current (*I*_*sc*_) in a concentration-dependent manner. Ion substitution of Cl^−^ or CFTR inhibitors NPPB and glibenclamide or a Na^+^/K^+^/2Cl^−^ cotransporter inhibitor bumetanide attenuated kaempferol-induced *I*_*sc*_ response. In permeabilized monolayers, selective channel inhibitors CFTRinh-172 and CaCCinh-A01 inhibited kaempferol-induced apical Cl^−^ current (*I*_*Cl*_), and K^+^ blockers BaCl_2_ and clotrimazole inhibited basolateral K^+^ current (*I*_*Kb*_). The kaempferol-induced *I*_*Cl*_ showed no additive effects with forskolin or 8cpt-cAMP. The kaempferol-induced *I*_*Cl*_ was mostly abolished by protein kinase A inhibitor H89, but not by tyrosine kinase inhibitors, AG490 and tyrphostin A23, or tyrosine phosphatase inhibitor vanadate. Treatment with kaempferol for 24 h increased the expression of CFTR protein as determined by the Western blot analysis. These results demonstrated that kaempferol activates Cl^−^ secretion across T84 cells by activating the apical Cl^−^ current and basolateral K^+^ current. The mechanisms may involve the cAMP/PKA pathway and CFTR expression. Taken together, these findings reveal the beneficial effects of kaempferol to increase fluid secretion which can be used to treat constipation.

## 1 Introduction

Colonic epithelial cells play an important role in controlling the balance between luminal absorption and secretion of ions and water. Epithelial Cl^−^ secretion establishes a crucial driving force for fluid secretion to prevent epithelial surface dehydration and bacteria/viral infection (Saint-Criq and Gray, 2017), and facilitates stool movement (Murek et al., 2010; Frizzell and Hanrahan, 2012). Increased Cl^−^ secretion helps to increase stool fluidity and relieve constipation. Conversely, reduced Cl^−^ secretion decreases fluid movement into the colonic lumen and can lead to constipation.

Cl^−^ secretion across an apical membrane of the colonic epithelial cells mainly occurs through two major types of Cl^−^ channels, cystic fibrosis transmembrane conductance regulator (CFTR), and Ca^2+^-activated Cl^−^ channel (CaCC). CFTR is a cAMP-dependent Cl^−^ channel mainly regulated by cAMP-dependent protein kinase A (PKA) and direct activation of CFTR via protein kinases (PKs)-mediated phosphorylation or protein phosphatases (PPs)-mediated dephosphorylation (Seibert et al., 1995; Baker and Hamilton, 2004). CaCC is directly controlled by cytoplasmic Ca^2+^ and indirectly via the interaction with Ca^2+^-binding protein calmodulin (Hawn et al., 2021). Moreover, basolateral transport proteins, Na^+^/K^+^-ATPase, Na^+^/K^+^/2Cl^−^ cotransporter (NKCC), and K^+^ channels are required to maintain a driving force for the process of Cl^−^ secretion (Matos et al., 2007).

According to studies in humans and rabbits, chronic constipation in elderly is associated with a decrease in cAMP-dependent Cl^−^ secretion and stool water content (Braaten et al., 1988; Veeze et al., 1994). Although evidence regarding decreased CFTR in the colon of elderly is still unclear, a study of single-cell transcriptomics in the lungs of aging mice reveals that CFTR is reduced in alveolar type II cells (Angelidis et al., 2019). The decrease in Cl^−^ secretion has been also found in airway epithelial cells of cystic fibrosis patients, which is caused by impairing cAMP-regulate Cl^−^ conductance (Itani et al., 2011). Conditions with high inflammatory mediators such as INF-γ and TNF-α are able to downregulate CFTR in colonic epithelial cells (Besancon et al., 1994). Hence, modulation of Cl^−^ secretion through mediating cAMP/PKA signaling pathway, direct activation of CFTR, or expression of CFTR could be the potential target for treating constipation.

Kaempferol a dietary flavonol has been demonstrated to stimulate electrogenic Cl^−^ secretion in T84 cells, but its effect is less than quercetin under basal conditions (Nguyen et al., 1991). However, a study has shown that kaempferol has the greatest effect on cAMP-activated Cl^−^ secretion by forskolin in human airway epithelium as compared to other flavonoids (Illek and Fischer, 1998). In addition, kaempferol classified as phytoestrogen may exert genomic effect in increased CFTR expression similar to phytoestrogen genistein (Schmidt et al., 2008). Therefore, we aimed to examine the pharmacological effects and cellular mechanisms of kaempferol on the regulation of ion transport and the expression of CFTR using T84 cells as the model of colonic epithelium (Dharmsathaphorn et al., 1984).

## 2 Materials and methods

### 2.1 Chemicals

Kaempferol, CFTR inhibitor-172 (CFTRinh-172), glibenclamide, 5-nitro-2-(3-phenylpropylamino) benzoic acid (NPPB), calcium-activated chloride channel inhibitor (CaCCinh-A01), 4,4′-Diisothiocyanatostilbene-2,2′-disulfonate (DIDS), forskolin, 1,2-bis(o-aminophenoxy)ethane-N,N,N′,N′-tetraacetic acid (BAPTA-AM), amphotericin B, H89, AG490, and tyrphostin A23 were dissolved in dimethyl sulfoxide (DMSO). The final concentration of DMSO was controlled at 0.01% in all tests. Amiloride, carbachol (CCh), 8-(4-chlorophenylthio) adenosine-3′,5′-cyclic monophosphate (8cpt-cAMP), and sodium orthovanadate (vanadate) were dissolved in distilled water. Bumetanide was dissolved in ethanol. The vehicles had no effect on *I*_*sc*_. All these chemicals were purchased from Sigma-Aldrich (MO, United States). Mouse anti-CFTR antibody, mouse anti-β-actin antibody, HRP-conjugated goat anti-mouse IgG antibody, and chemiluminescence ECL were obtained from Santa Cruz Biotechnology (CA, United States). Bicinchoninic acid assay (BCA assay) was purchased from Visual Protein (TW, ROC). Laemmli loading buffer and mercaptoethanol were purchased from Bio-rad (CA, United States).

### 2.2 Cell culture

Human colonic adenocarcinoma cell line (T84 cells) purchased from the American Type Culture Collection (VA, United States), were plated on a 100-mm cell culture dish. The T84 cells (passage number 61–86) were cultured in standard media composed of DMEM-Ham’s F-12 (DMEM-F12; 1:1) with 10% fetal bovine serum, and 1% penicillin-streptomycin at 37°C in 5% CO_2_ incubator. Cell culture reagents and supplies were purchased from Gibco (NY, United States). Culture media were refreshed every 2 days. When the cells reached 80% confluence, they were detached using trypsin-EDTA (0.25%) and transferred into new suitable cell culture vessels.

### 2.3 Cytotoxicity effects of kaempferol on T84 cells

Cytotoxicity effects of kaempferol on T84 cells were determined using the MTT colorimetric assay. The cells were seeded in a 48-well plate at 1 × 10^5^ cells/well and incubated for 7 days. Afterward, the cells were treated with various concentrations of kaempferol (1, 5, 10, 50, and 100 µM) in standard media for 24 and 48 h. An equivalent quantity of DMSO was used as vehicle control. 10% MTT solution in media (125 µL) was added to each well and incubated in a CO_2_ incubator for 3 h. The MTT-derived formazan crystals were dissolved in 100 µL of DMSO, and the absorbance was measured in optical density (OD) unit at wavelength of 570 nm and 620 nm using a microplate reader (Epoch, Biotek, VM, United States). OD unit of kaempferol at 570 nm subtraction with OD unit at 620 nm was calculated for cytotoxicity analysis by comparing with those of the vehicle DMSO. The experiments were conducted in duplicate and repeated at least three times for each concentration of kaempferol.

### 2.4 Measurement of electrical parameters

T84 cells (3 × 10^5^ cells/well) were cultured into the Snapwell insert with a 0.4 µm pore polycarbonate membrane (Costar, MA, United States) for 10–14 days to form the complete monolayer detected by assessing Transepithelial electrical resistance (TEER) using a Millicell ERS-2 volt-ohm meter coupled to Ag/AgCl electrodes (World Precision Instrument, FL, United States). The monolayers with high resistance (1,500–3,000 Ω cm^2^) were selected for Ussing experiments. The monolayers were mounted in Ussing chambers bathed with standard Ringer solution pH 7.4 (in mM: 118 NaCl, 4.5 KCl, 2.5 CaCl_2_, 0.54 MgCl_2_, 25 NaHCO_3_, 1.5 NaH_2_PO_4_). The solution was bubbled with 95% O_2_ and 5% CO_2_ (carbogen) and maintained at 37°C. Short-circuit currents (*I*_*sc*_) and transepithelial potential difference (PD) were measured by an EVC-4,000 voltage/current clamp (World Precision Instrument, United States) with Ag/AgCl electrodes connected to bathing solution via agar bridges. Transepithelial conductance (G) was calculated using Ohm’s law (G = *I*_*sc*_/PD). The monolayers were short-circuited, except for measuring the PD value before and after adding test substances. The voltage clamp data was sent through a PowerLab 4/35 A/D converter and recorded on an Intel® Core™ i5-3,570, 3.40 GHz, 3,401 MHz Processors. The monolayers were equilibrated for at least 30 min. Using an apical side as a reference, a positive *I*_*sc*_ indicated a net flux of positive charges from the apical side to the basolateral side (absorptive direction) or net flux of negative charges from the basolateral side to the apical side (secretory direction). In anion substitution experiments, *I*_*sc*_ was measured from monolayers bathed with Cl^−^ free, HCO_3_^−^-free or Cl^−^ and HCO_3_^−^-free Ringer solution. In Cl^−^ free solution, gluconate salts were used to replace Cl^−^, and bubbled with carbogen, while in HCO_3_^−^ free solution, HEPES buffer was used to replace HCO_3_^−^. Both HCO_3_^−^ free and Cl^−^-HCO_3_^−^ free Ringer solutions were bubbled with 100% O_2_.

Membrane permeability studies were performed using amphotericin B-permeabilized monolayer. To determine apical Cl^−^ current (*I*_*Cl*_), the apical side was filled with a high concentrated KCl Ringer solution pH 7.4 (in mM: 111.5 KCl, 25 NaHCO_3_, 12 D-glucose, 1.8 Na_2_HPO_4_, 1.25 CaCl_2_, 1 MgSO_4_, 0.2 NaH_2_PO_4_) and the basolateral side filled with a KMeSO_4_ Ringer solution pH 7.4 (in mM: 120 KMeSO_4_, 20 KHCO_3_, 15 mannitol, 5 NaCl, 2 calcium gluconate, 1.3 K_2_HPO_4_, 1 MgSO_4_, 0.3 KH_2_PO_4_). Amphotericin B (50 µM) was added into basolateral solution for basolateral membrane permeabilization. Basolateral K^+^ current (*I*_*Kb*_) was measured when the apical side was filled with KMeSO_4_ Ringer solution in the presence of amphotericin B, while the basolateral side was filled with a NaMeSO_4_ Ringer solution pH 7.4 (in mM: 120 NaMeSO_4_, 30 mannitol, 5 NaCl, 3 calcium gluconate, 1 MgSO_4_, 20 KHCO_3_, 0.3 KH_2_PO_4_, 1.3 K_2_HPO_4_). All solutions were maintained at 37°C and bubbled with carbogen.

### 2.5 CFTR protein expression by Western blot analysis

The effect of kaempferol on the CFTR protein expression was tested by Western blot analysis. T84 cells (3 × 10^5^ cells/well) were cultured in 60-mm culture dishes and then treated with either 50 µM or 100 µM of kaempferol in standard media or DMSO alone for 24 h. Total proteins were extracted from the cells by a lysis buffer containing (in mM) 46.5 Tris (adjust pH 7.4), 150 mM NaCl, 1 EDTA, 1 NaF, 1 PMSF, 1% NP-40, and 1% protease inhibitor mixture (Sigma, United States) and measured concentrations by the Bicinchoninic acid assay (BCA assay). Protein samples (60 μg/mL) were loaded into 7.5% Sodium Dodecyl Sulfate polyacrylamide gel electrophoresis (SDS-PAGE), run at 100 V for 120 min, and subsequently transferred to a polyvinylidene difluoride (PVDF) membrane (Millipore, United States). The membrane was blocked with 5% non-fat milk for 1 h before incubation at 4°C overnight with primary antibodies, i.e., mouse monoclonal anti-CFTR (1:100 dilution), and anti-β-actin (1:1,000 dilution) and further incubated at room temperature for 1 h with horseradish peroxidase-conjugated goat anti-mouse IgG secondary antibody (1:2,000 dilution). Immunoreactive signals were visualized by the ECL substrate and imaged by the ChemiDoc Imaging System (Bio-Rad, United States). The protein expression of CFTR was analyzed by ImageJ (NIH, United States) and normalized to β-actin. All experiments were replicated at least three times to verify reproducibility.

### 2.6 Statistical analysis

Data were expressed as mean ± standard deviation; n referred to the number of independent experiments from different passages in each group. Statistical differences between mean values of the control and one treated group were analyzed by Student’s t-test. To compare cytotoxic effect of different treatments on different times, two-way analysis of variance (two-way ANOVA) was performed followed by Dunnett’s *post-hoc* test for comparison with control. Analysis of variance (ANOVA) followed by Tukey’s *post-hoc* test were used to compare the various concentration effect of drugs. All statistics were analyzed using GraphPad Prism software version 8.0.2, Inc., San Diego, CA. A value of *P* < 0.05 was considered statistically significant.

## 3 Results

### 3.1 Cytotoxic effect of kaempferol

**FIGURE 1:**
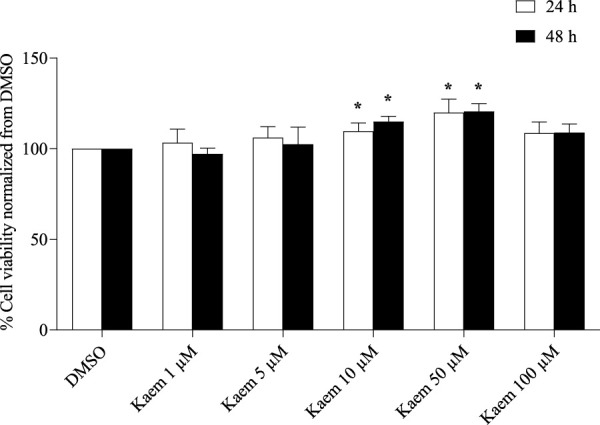
The cytotoxicity of kaempferol in T84 cells. Cells exposed to 1–100 µM of kaempferol for 24 and 48 h were evaluated for cell viability using the MTT assay. Data are represented as mean ± SD (n = 3–5) of % cell viability relative to the DMSO control. *P < 0.05 compared with DMSO at specific incubation times by two-way ANOVA and Dunnett’s *post-hoc* test. No significant difference in % cell viability was observed between 24 and 48 h for each treatment as analyzed by two-way ANOVA and followed by Dunnett’s post-hoc test.

Cytotoxic effect of kaempferol treatment at concentrations of 1, 5, 10, 50, and 100 µM for 24 or 48 h on T84 cells was determined by the MTT assay. The results showed that all treatments with kaempferol for 24 or 48 h had no toxic effects to T84 cells as compared with the corresponding DMSO control (Figure 1). Conversely, the cell viability was proportionally increased with kaempferol concentrations from 1 to 50 µM. Treatment with kaempferol 10 and 50 µM for 24 and 48 h significantly increased T84 cell viability compared with the corresponding DMSO (n = 3–5, *P* < 0.05, Figure 1). However, the kaempferol treatments at the same concentration did not significantly change cell viability between 24 and 48 h.

### 3.2 Effect of kaempferol on basal *I*_*sc*_

**FIGURE 2:**
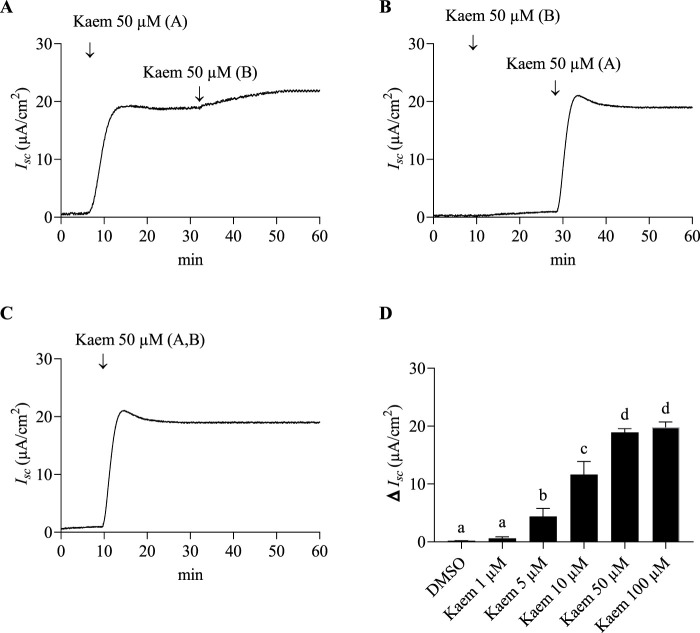
Effect of kaempferol on basal *I*_*sc*_ response in T84 cell monolayers. Representative *I*_*sc*_ tracings in response to **(A)** the apical addition of kaempferol (50 µM) followed by basolateral addition (n = 4) **(B)** the basolateral addition of kaempferol followed by apical addition (n = 4). **(C)** kaempferol (50 μM, apical and basolateral) (n = 8). **(D)** Average changes in *I*_*sc*_ response induced by kaempferol at concentrations ranging from 1 to 100 µM (apical and basolateral) (n = 4). Values are expressed as mean ± SD (n = 4). Bar graph with different letters indicates significantly different among groups at *P* < 0.05 by one-way ANOVA and Tukey’s *post-hoc* test. A = apical; B = basolateral.

Under basal conditions, the average *I*_*sc*_, PD, and G values of T84 cell monolayers mounted with standard Ringer solution in Ussing chambers were 1.18 ± 0.66 μA/cm^2^, −1.96 ± 1.05 mV, and 0.62 ± 0.18 mS/cm^2^ (n = 102), respectively. We first investigated the effect and route of kaempferol at the effective concentration of 50 µM from previous study on basal *I*_*sc*_. We found that kaempferol 50 µM increased *I*_*sc*_ when added to the apical side and slightly increased *I*_*sc*_ after a subsequent addition to the basolateral side (n = 4, Figure 2A). Meanwhile, a basolateral addition of kaempferol led to a small increase in *I*_*sc*_, and a subsequent apical addition significantly increased *I*_*sc*_ (n = 4, Figure 2B). This indicates the apical membrane is a major site of kaempferol action. To ensure the maximum effect on *I*_*sc*_, kaempferol at each concentration was applied to both apical and basolateral solution.

Bilateral addition of kaempferol (50 µM) induced an increase in *I*_*sc*_ within 7–8 min to a maximal response of 19.54 ± 4.46 μA/cm^2^ (n = 8, Figure 2C), then decreased slightly and maintained continuously up to 90 min. A single dose of kaempferol varying from 1 to 100 µM was additionally tested for dose-dependent response. As shown in Figure 2D, kaempferol 10, 50, and 100 µM significantly increased the *I*_*sc*_ as compared with DMSO (n = 4, *P* < 0.0001) with a maximum activation at 50 and 100 µM. Thus, kaempferol 50 μM, an observed minimum concentration that produced the highest *I*_*sc*_ activation, was selected for the remaining experiments.

### 3.3 Effect of Cl^−^ channel blockers and bumetanide on kaempferol-induced *I*_*sc*_

**FIGURE 3:**
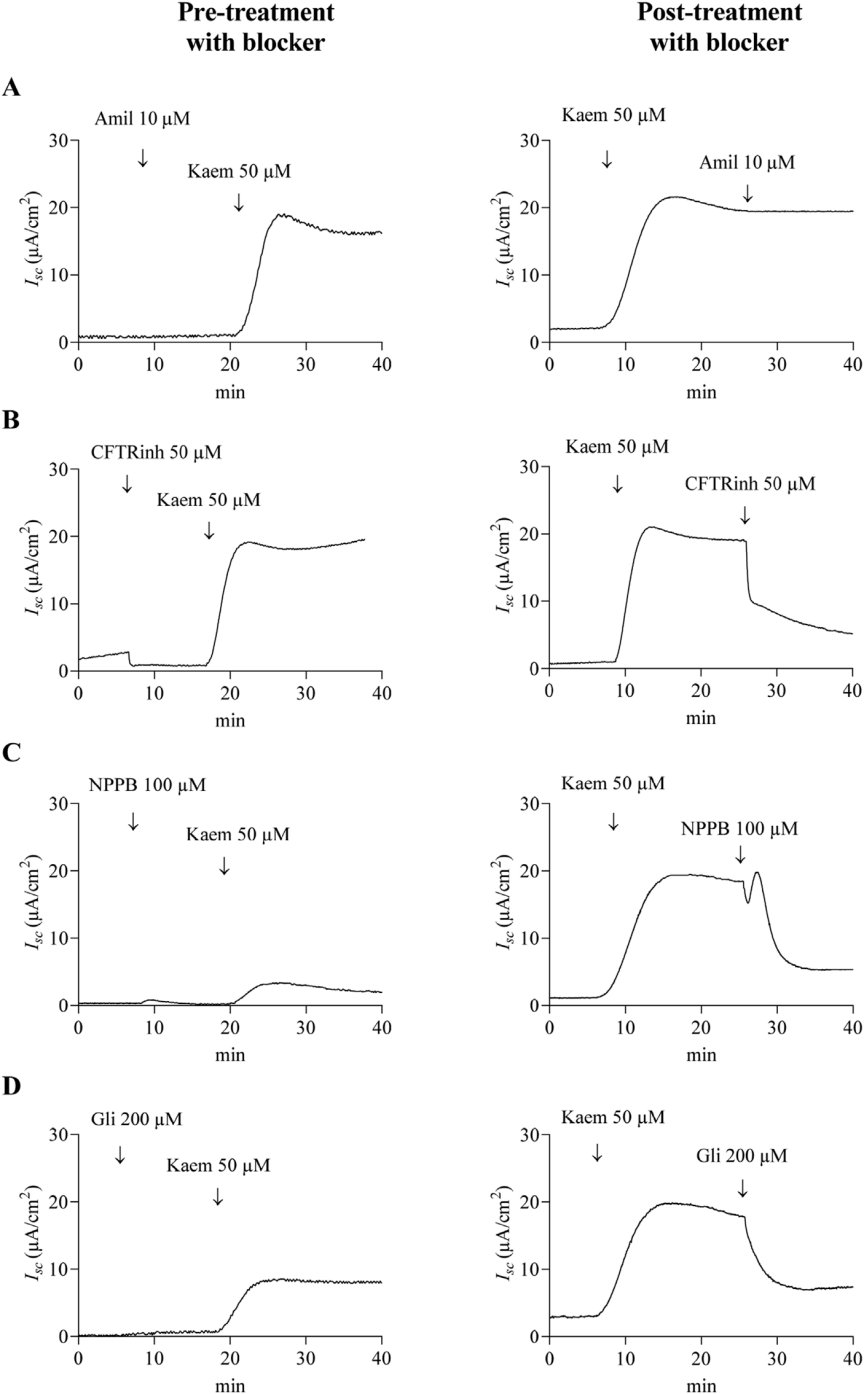
Effect of Na^+^ and CFTR Cl^−^ channel blockers on kaempferol-activated *I*_*sc*_ in T84 cell monolayers. Representative tracings of *I*_*sc*_ induced by kaempferol (50 μM, apical and basolateral) after pretreatment or posttreatment with **(A)** Na^+^ channel blocker amiloride (10 μM, apical), or **(B–D)** CFTR Cl^−^ channel blockers **(B)** CFTRinh-172 (50 μM, apical), **(C)** NPPB (100 μM, apical), or **(D)** glibenclamide (200 μM, apical).

As kaempferol increased *I*_*sc*_ response in T84 cell monolayers, we further investigated the ionic basis of the kaempferol-induced *I*_*sc*_ by applying various pharmacological channel blockers in apical solution before (pretreatment) and after (posttreatment) kaempferol addition. As shown in Figure 3, kaempferol-induced increase in *I*_*sc*_ was not inhibited by amiloride (10 µM), the Na^+^ channel blocker, whether added before or after kaempferol (Figures 3A, 5A,B). Pretreatment of CFTRinh-172 (50 µM), the selective CFTR inhibitor, failed to inhibit the kaempferol-increased *I*_*sc*_ (Figures 3B, 5A) whereas posttreatment of CFTRinh-172 decreased the *I*_*sc*_ by 53% from the maximum effect of kaempferol (−10.29 ± 2.48 μA/cm^2^, n = 8, Figures 3B, 5B). Conversely, the kaempferol response was significantly inhibited by pretreatment with 5-Nitro-2-(3-phenylpropylamino) benzoic acid (NPPB) (100 µM) or glibenclamide (200 µM), CFTR inhibitors by 84% (3.09 ± 0.23 μA/cm^2^, n = 4, *P* < 0.05, Figures 3C, 5A) or 64% (7.06 ± 1.15 μA/cm^2^, n = 4, *P* < 0.05, Figures 3D, 5A), respectively, as compared with kaempferol alone. The posttreatment with NPPB or glibenclamide decreased the kaempferol response by 60% (−11.79 ± 1.82 μA/cm^2^, n = 4, *P* < 0.05, Figures 3C, 5B) or 58% (−11.33 ± 0.96 μA/cm^2^, n = 4, *P* < 0.05, Figures 3D, 5B), respectively.

**FIGURE 4:**
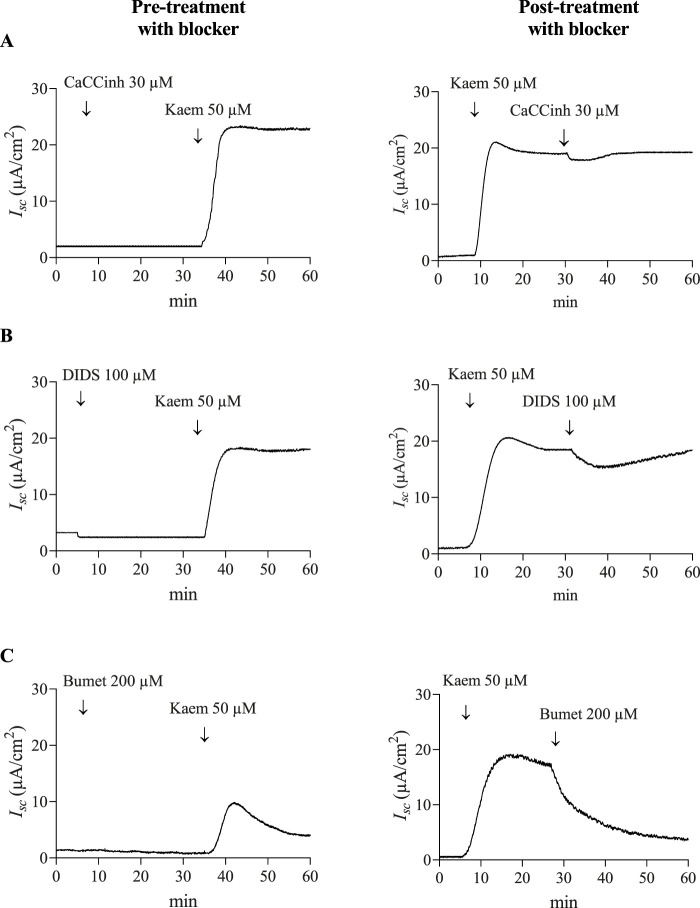
Effect of CaCC and NKCC cotransporter blockers on kaempferol-activated *I*_*sc*_ in T84 cell monolayers. Representative tracings of *I*_*sc*_ induced by kaempferol (50 μM, apical and basolateral) after pretreatment or posttreatment with CaCC blockers **(A)** CaCCinh-A01 (30 μM, apical), **(B)** DIDS (100 μM, apical), or **(C)** NKCC cotransporter blocker bumetanide (200 μM, basolateral).

**FIGURE 5:**
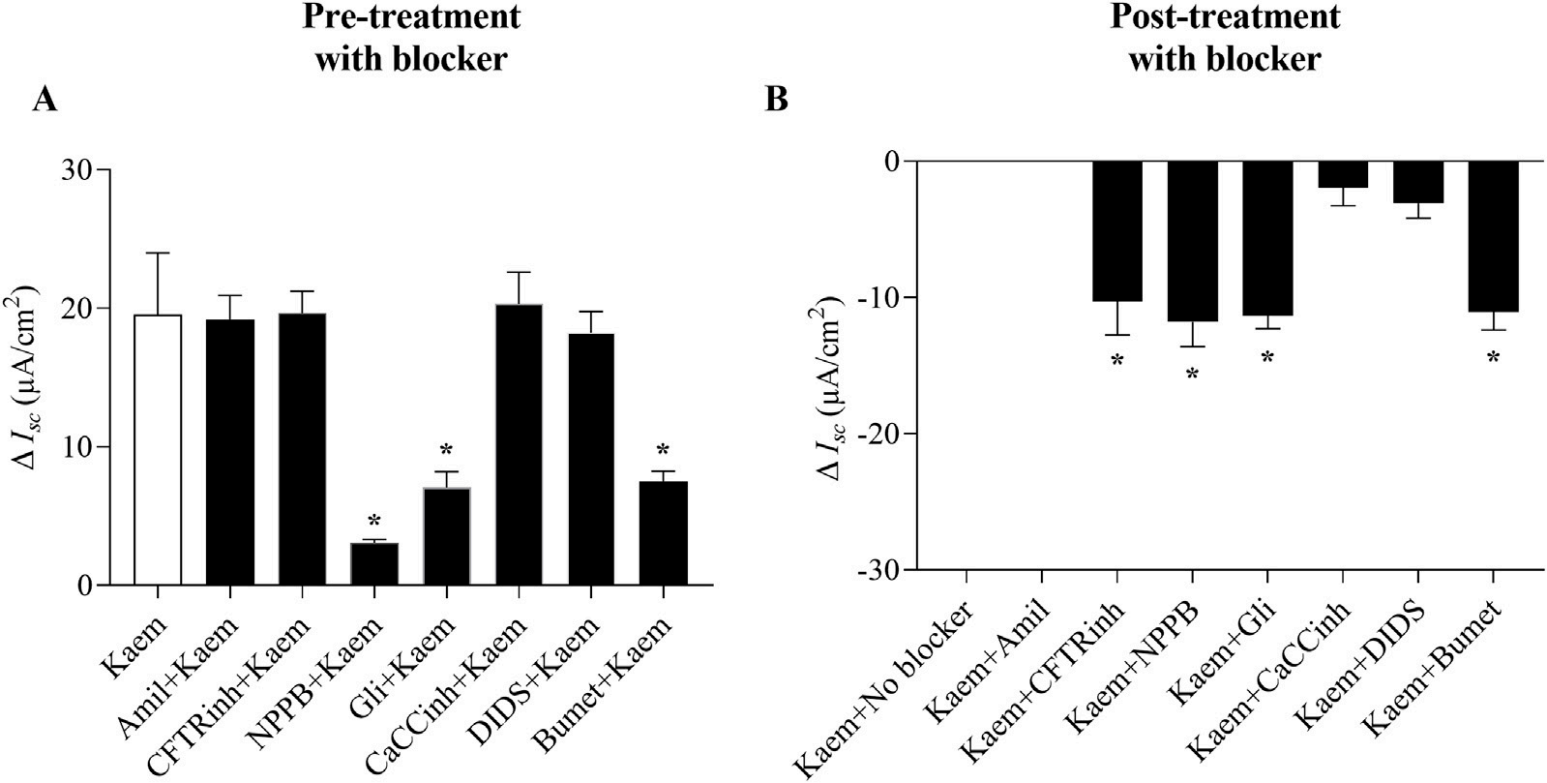
Effect of channel and cotransporter blockers on kaempferol-activated *I*_*sc*_ in T84 cell monolayers. **(A)** Bar graph showing average changes in *I*_*sc*_ induced by kaempferol alone or after pretreatment with Na^+^ channel, CFTR, CaCC, or NKCC blockers. **(B)** Bar graph showing average changes in the inhibition of kaempferol-induced *I*_*sc*_ from maximum after posttreatment with these blockers. Values are expressed as mean ± SD (n = 4–8). *P < 0.05 compared with kaempferol alone **(A)** or kaempferol with no blocker **(B)** by one-way ANOVA and Dunnett’s *post-hoc* test.

Our study also showed that pretreatment with CaCCinh-A01 (30 µM) or DIDS (100 µM), CaCC inhibitors, failed to inhibit the kaempferol-induced increase in *I*_*sc*_ (n = 4, Figures 4A,B, 5A). Nevertheless, posttreatment with CaCCinh-A01 or DIDS decreased the *I*_*sc*_ from the maximum effect of kaempferol by 10% (−1.92 ± 1.35 μA/cm^2^, n = 5, Figures 4A, 5B) or 16% (−3.10 ± 1.08 μA/cm^2^, n = 5, Figures 4B, 5B), respectively. Besides, pretreatment with bumetanide (200 µM) in basolateral solution to block the Cl^−^ uptake through NKCC cotransporter reduced the kaempferol-induced *I*_*sc*_ by 61% (7.53 ± 0.73 μA/cm^2^, n = 4, *P* < 0.05, Figures 4C, 5A), and posttreatment decreased the *I*_*sc*_ from maximum kaempferol response by 58% (−11.05 ± 1.34 μA/cm^2^, n = 4, Figures 4C, 5B). Taken together, our findings indicate kaempferol stimulation of anion secretion, albeit with little active CFTR.

**FIGURE 6:**
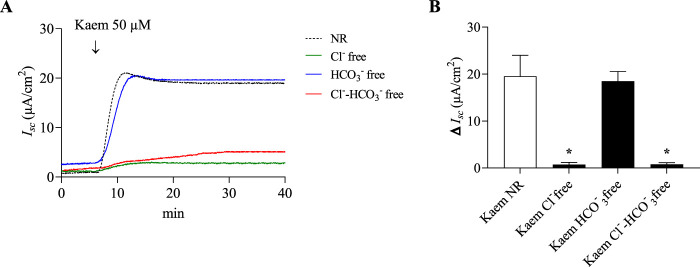
Effect of Cl^−^ or HCO_3_^−^ substitution on kaempferol-activated *I*_*sc*_ in T84 cell monolayers. **(A)** Representative *I*_*sc*_ tracings induced by kaempferol (50 μM, apical and basolateral) in normal Ringer (NR), Cl^−^ free, HCO_3_^−^ free, or Cl^−^-HCO_3_^-^ free solutions. **(B)** Bar graph showing average changes in the kaempferol *I*_*sc*_ response in these solutions. Values are expressed as mean ± SD (n = 4). *P < 0.05 compared with kaempferol alone by one-way ANOVA and Dunnett’s *post-hoc* test.

Anion substitution tests were additionally carried out to confirm the ionic basis of the kaempferol effect on anion secretion. As shown in Figures 6A,B, adding kaempferol to normal Ringer solution increased the *I*_*sc*_ by 19.54 ± 4.46 μA/cm^2^ (n = 8). The kaempferol-induced *I*_*sc*_ was unaffected when HCO_3_^−^ was substituted (18.49 ± 2.07 μA/cm^2^, n = 4), but it was significantly decreased by 96% in Cl^−^ free (0.73 ± 0.45 μA/cm^2^, n = 4, *P* < 0.05) or Cl^−^ and HCO_3_^−^ free solution (0.78 ± 0.35 μA/cm^2^, n = 4, *P* < 0.05). This indicates that the kaempferol-stimulated *I*_*sc*_ is Cl^−^ dependent.

### 3.4 Effect of kaempferol on forskolin and carbachol-activated *I*_*sc*_

**FIGURE 7:**
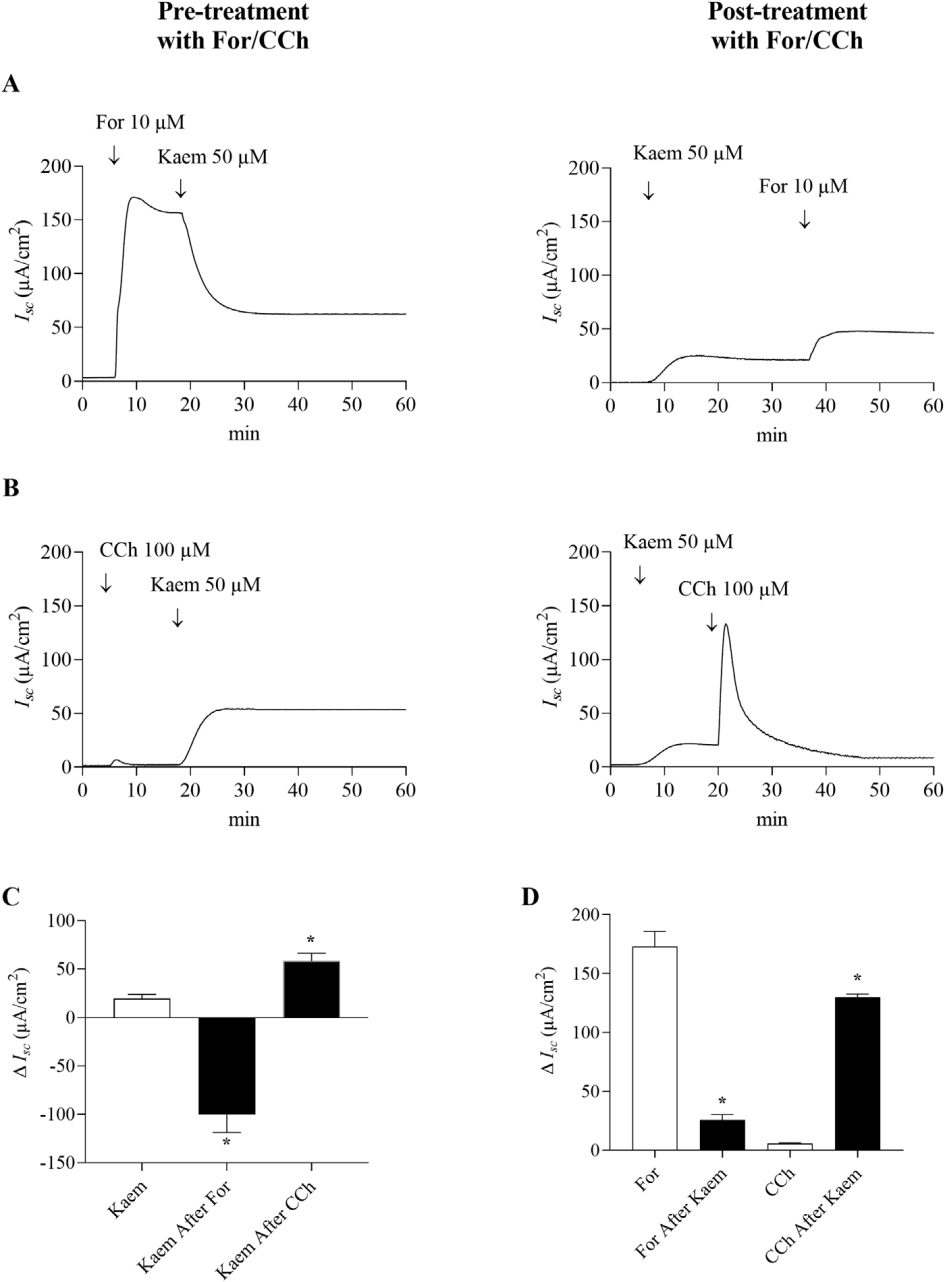
Effect of kaempferol on forskolin and carbachol-activated transepithelial Cl^−^ secretion in T84 cell monolayers. Representative *I*_*sc*_ tracings induced by kaempferol (50 μM, apical and basolateral) after pretreatment or posttreatment with **(A)** forskolin (10 μM, apical and basolateral) or **(B)** carbachol (100 μM, basolateral). **(C)** Bar graph showing average changes in *I*_*sc*_ induced by kaempferol alone and after pretreatment with forskolin or carbachol, or **(D)** changes in *I*_*sc*_ induced by forskolin or carbachol alone and after pretreatment with kaempferol. Values are expressed as mean ± SD (n = 4). *P < 0.05 compared with kaempferol alone by one-way ANOVA and Dunnett’s *post-hoc* test **(C)**, or *P < 0.05 compared with forskolin and carbachol alone by Student’s t-test **(D)**.

The involvement of intracellular cAMP or Ca^2+^ in kaempferol-sensitive *I*_*sc*_ was further investigated under Cl^−^ secretion activated by forskolin or carbachol. The kaempferol-sensitive *I*_*sc*_ was determined before and after addition of forskolin or carbachol as shown in Figure 7. Addition of forskolin (10 μM, bilateral) induced the maximal *I*_*sc*_ response to 172.91 ± 12.89 μA/cm^2^ (n = 4, Figures 7A,D). A subsequent addition of kaempferol decreased the forskolin-stimulated *I*_*sc*_ by 55% (−94.42 ± 15.57 μA/cm^2^, n = 4, Figures 7A,C). Pretreatment with kaempferol increased *I*_*sc*_ and reduced the forskolin-activated *I*_*sc*_ to 25.81 ± 4.52 μA/cm^2^ (n = 4), which was less than the *I*_*sc*_ induced by forskolin alone by 85% (Figures 7A,D). To determine the kaempferol effect on Ca^2+^-activated Cl^−^ secretion, it was shown in Figure 7B that carbachol (100 μM, basolateral) increased *I*_*sc*_ by 5.87 ± 0.55 μA/cm^2^ (n = 4). Subsequent addition of kaempferol further increased the *I*_*sc*_ response to 58.34 ± 8.05 μA/cm^2^ (n = 4), which was three times greater than the kaempferol alone (Figures 7B,C). However, pretreatment with kaempferol greatly increased the maximal *I*_*sc*_ response induced by carbachol to 129.92 ± 2.74 μA/cm^2^ (n = 4, Figures 7B,D).

### 3.5 Effect of kaempferol on membrane permeability

**FIGURE 8:**
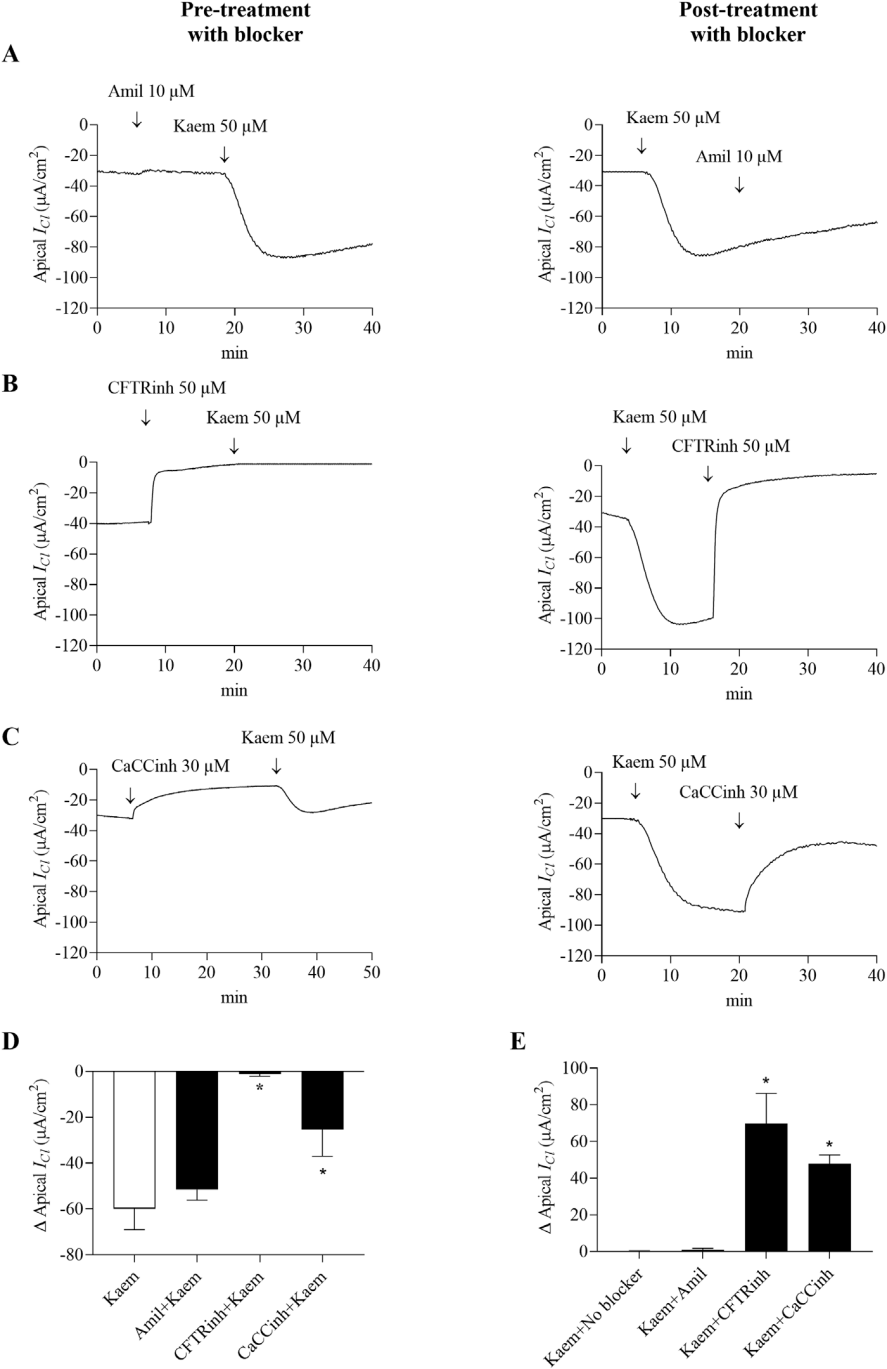
Effect of kaempferol on apical Cl^−^ current (*I*_*Cl*_) in the basolaterally permeabilized T84 cell monolayers. Representative tracings of apical *I*_*Cl*_ induced by kaempferol (50 μM, apical and basolateral) after pretreatment or posttreatment with **(A)** Na^+^ channel blocker amiloride (10 μM, apical), or selective Cl^−^ channel blockers **(B)** CFTRinh-172 (50 μM, apical), or **(C)** CaCCinh-A01 (30 μM, apical). Bar graph showing **(D)** average changes in apical *I*_*Cl*_ response induced by kaempferol alone and after pretreatment with these blockers. Values are expressed as mean ± SD (n = 4). *P < 0.05 compared with kaempferol alone by one-way ANOVA and Dunnett’s *post-hoc* test. **(E)** Average changes in the inhibition of kaempferol-induced apical *I*_*Cl*_ from maximum after posttreatment with these blockers. Values are expressed as mean ± SD (n = 4). *P < 0.05 compared with kaempferol no blocker by one-way ANOVA and Dunnett’s *post-hoc* test.

The basolateral membrane permeabilized monolayers were used to examine the effect of kaempferol on apical Cl^−^ permeability. Bilateral addition of kaempferol maximally increased *I*_*Cl*_ by −59.83 ± 9.20 μA/cm^2^ (n = 6, Figures 8A,D), as shown by the negative deflection of the current. Like the *I*_*sc*_, either pretreatment or posttreatment with amiloride (10 μM, apical) did not inhibit the stimulatory effect of kaempferol on the *I*_*Cl*_. The kaempferol-stimulated *I*_*Cl*_ after amiloride was −51.56 ± 4.62 μA/cm^2^, n = 4, Figures 8A,D). In contrast, apical pretreatment of CFTRinh-172 (50 µM) or CaCCinh-A01 (30 µM) inhibited the kaempferol-induced increase in *I*_*Cl*_ by 98% (−1.13 ± 0.95 μA/cm^2^, n = 4, *P* < 0.05, Figures 8B–D) and 58% (−25.29 ± 11.74 μA/cm^2^, n = 4, *P* < 0.05, Figures 8C,D), respectively. Posttreatment with CFTRinh-172 completely inhibited the kaempferol response and basal *I*_*Cl*_ which accounted for 117% (69.89 ± 16.36 μA/cm^2^, n = 4, *P* < 0.05, Figures 8B,E) and CaCCinh-A01 markedly inhibited 80% from the maximum kaempferol response (47.84 ± 4.85 μA/cm^2^, n = 4, *P* < 0.05, Figures 8C,E).

**FIGURE 9:**
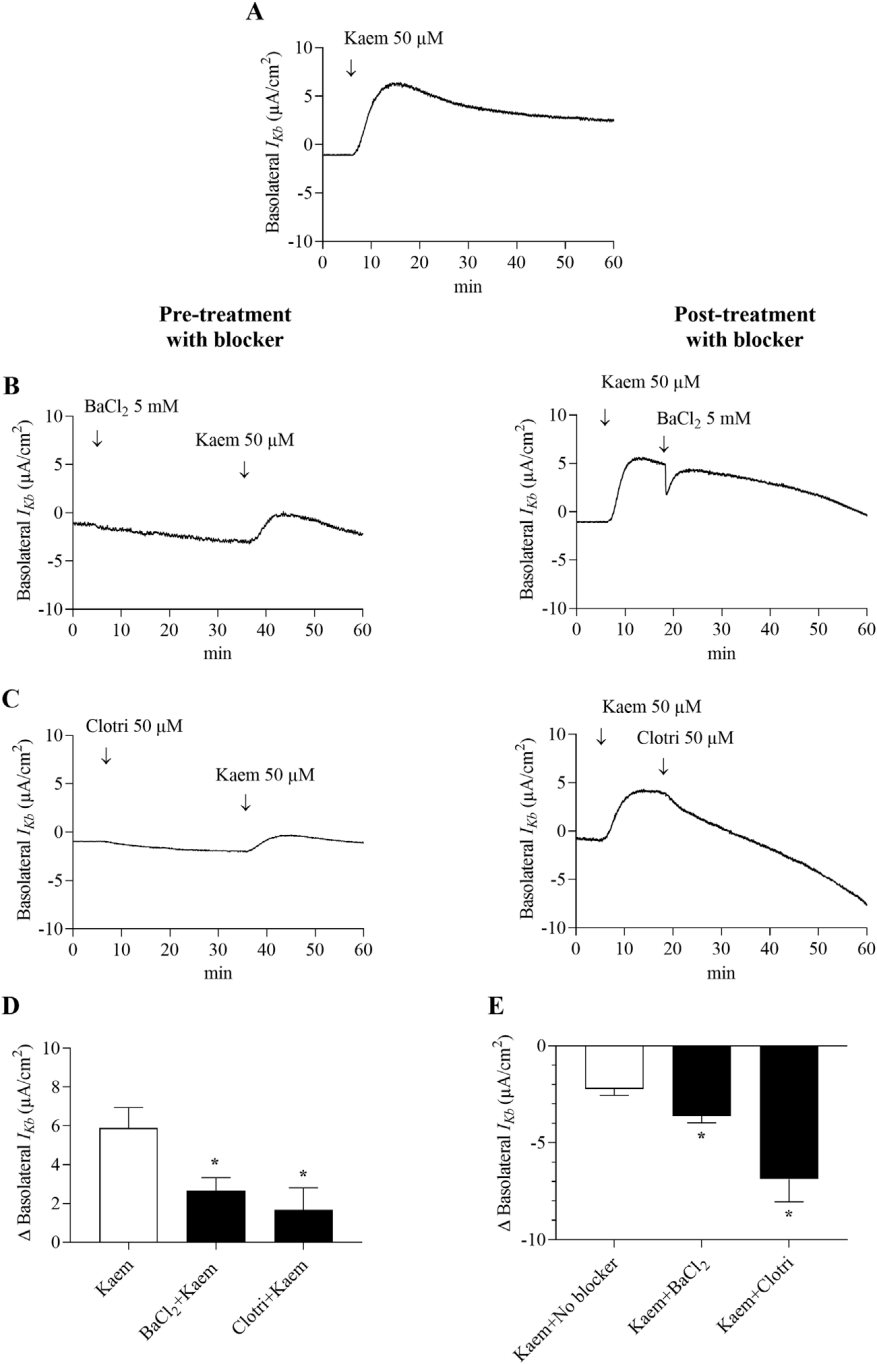
Effect of kaempferol on basolateral K^+^ current (*I*_*Kb*_) in the apically permeabilized T84 cell monolayers. Representative tracing of **(A)** basolateral *I*_*Kb*_ induced by kaempferol (50 μM, apical and basolateral) alone (n = 12) and kaempferol after basolateral pretreatment or posttreatment with **(B)** BaCl_2_ (5 mM) or **(C)** clotrimazole (50 µM). Bar graph showing **(D)** average changes in basolateral *I*_*Kb*_ response induced by kaempferol alone and after pretreatment with BaCl_2_ or clotrimazole. Values are expressed as mean ± SD (n = 5). *P < 0.05 compared with kaempferol alone by one-way ANOVA and Dunnett’s *post-hoc* test. **(E)** Average changes in the inhibition of kaempferol-induced basolateral *I*_*Kb*_ from maximum after posttreatment with BaCl_2_ or clotrimazole. Values are expressed as mean ± SD (n = 4). *P < 0.05 compared with kaempferol no blocker by one-way ANOVA and Dunnett’s *post-hoc* test.

In the apical membrane-permeabilized monolayer, kaempferol at 50 µM increased the maximum *I*_*Kb*_ by 5.89 ± 1.05 μA/cm^2^ within 7–8 min, as shown by the positive deflection of the current, and then declined slightly (n = 12, Figure 9A). Basolateral pretreatment with a non-specific K^+^ channel blocker, BaCl_2_ (5 mM), and a Ca^2+^ activated K^+^ channel blocker, clotrimazole (50 µM) significantly inhibited the kaempferol-activated *I*_*Kb*_ by 55% (2.66 ± 0.67 μA/cm^2^, n = 5, *P* < 0.05, Figures 9B,D) and by 72% (1.67 ± 1.14 μA/cm^2^, n = 5, *P* < 0.05, Figures 9C,D), respectively. Posttreatment with BaCl_2_ and clotrimazole inhibited the maximum kaempferol response by 62% (−3.63 ± 0.34 μA/cm^2^, n = 4, *P* < 0.05, Figures 9B,E) and by 117% (−6.88 ± 1.16 μA/cm^2^, n = 4, *P* < 0.05, Figures 9C,E), respectively. These findings demonstrate that kaempferol activates Cl^−^ secretion mainly through CFTR and partially through CaCC and basolateral K^+^ channels.

### 3.6 Effect of kaempferol on cAMP and Ca^2+^-activated Cl^−^ current

**FIGURE 10:**
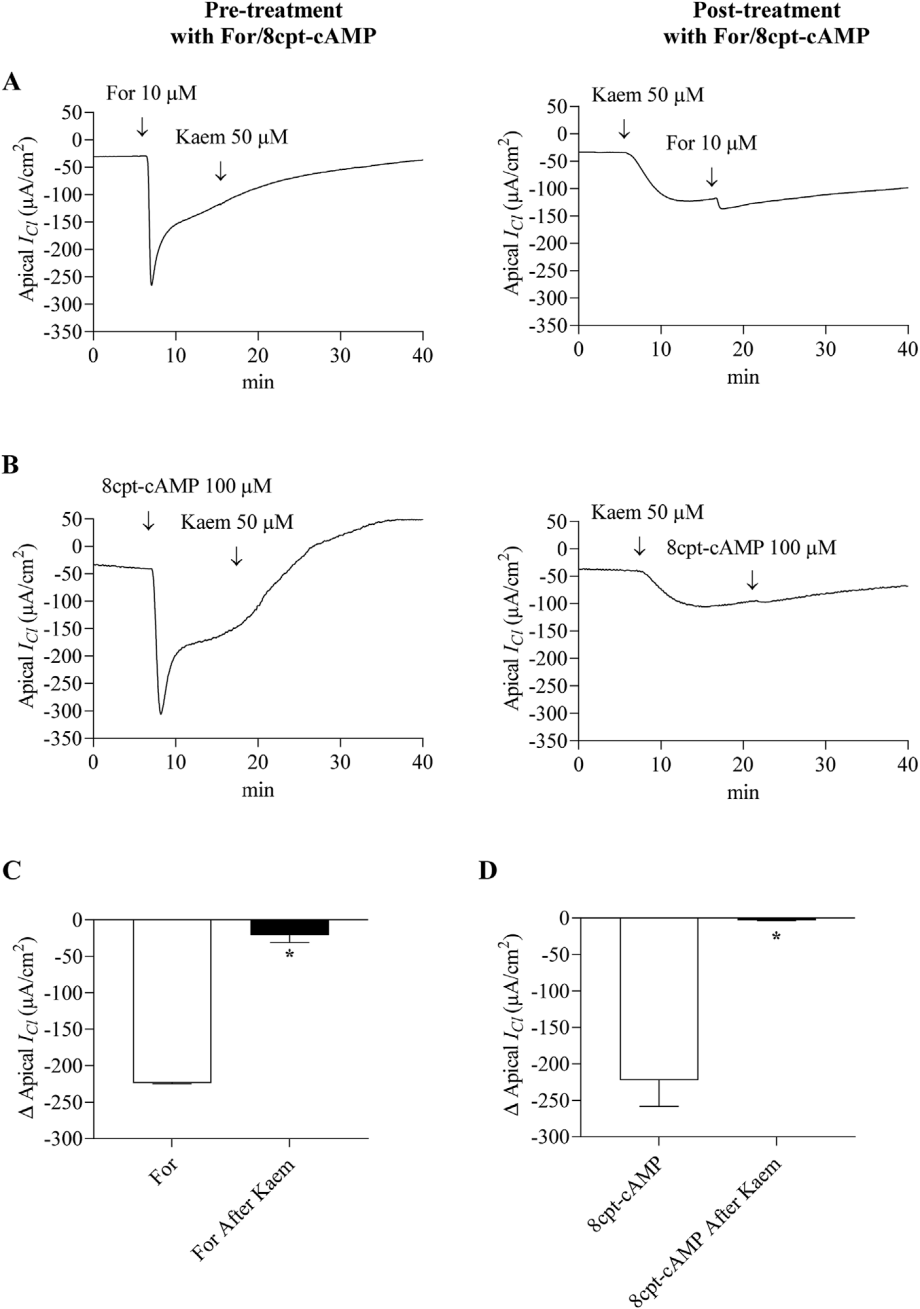
Effect of kaempferol on forskolin and 8cpt-cAMP-activated Cl^−^ current in the basolaterally permeabilized T84 monolayers. Representative tracings of apical *I*_*Cl*_ induced by kaempferol (50 μM, apical and basolateral) after pretreatment or posttreatment with **(A)** forskolin (10 μM, apical and basolateral) or **(B)** 8cpt-cAMP (100 μM, basolateral). Bar graph showing average changes in apical *I*_*Cl*_ responding to **(C)** forskolin alone or **(D)** 8cpt-cAMP alone and after pretreatment with kaempferol. Values are expressed as mean ± SD (n = 4). *P < 0.05 compared with forskolin alone or 8cpt-cAMP alone by Student’s t-test.

To verify whether cAMP plays a role in the kaempferol response, the kaempferol-activated *I*_*Cl*_ was determined before and after adding forskolin or 8cpt-cAMP which stimulated Cl^−^ secretion through CFTR. As shown in Figure 10, addition of forskolin (10 μM, apical and basolateral) or 8cpt-cAMP (100 μM, basolateral) activated the maximal *I*_*Cl*_ response by −223.85 ± 1.08 μA/cm^2^ (n = 4, Figures 10A,C) or −222.30 ± 35.90 μA/cm^2^ (n = 4, Figures 10B,D), respectively. The subsequent addition of kaempferol did not affect the forskolin or 8cpt-cAMP-stimulated *I*_*Cl*_ response pattern (Figures 10A,B). Conversely, pretreatment with kaempferol increased the *I*_*Cl*_ and decreased the forskolin or 8cpt-cAMP-activated *I*_*Cl*_ to −21.19 ± 9.97 μA/cm^2^ (n = 4, Figures 10A,C) or −3.01 ± 0.74 μA/cm^2^ (n = 4, Figures 10B–D), respectively, which was less than forskolin alone by 91% or 8cpt-cAMP alone by 99%.

**FIGURE 11:**
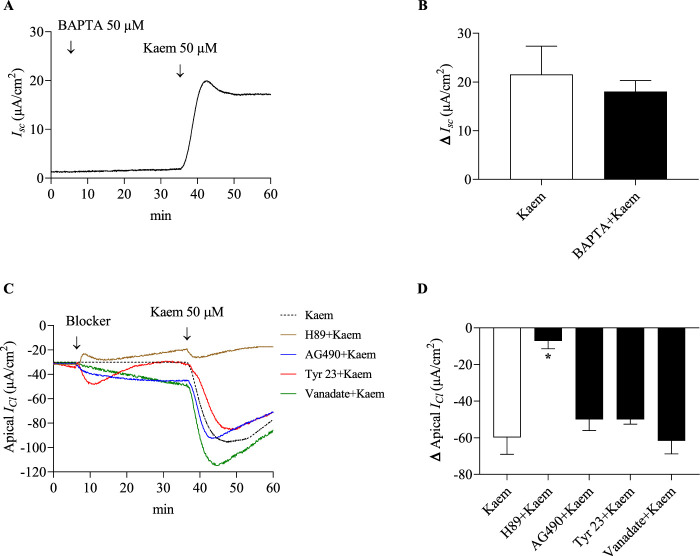
Effect of Ca^2+^ chelating BAPTA-AM on kaempferol-activated *I*_*sc*_ and effect of protein kinase A inhibitor, tyrosine kinase inhibitors, and tyrosine phosphatase inhibitor on kaempferol-activated *I*_*Cl*_ in permeabilized T84 monolayers. **(A)** A representative *I*_*sc*_ tracing induced by kaempferol (50 μM, apical and basolateral) after pretreatment with BAPTA-AM (50 μM, apical and basolateral). **(B)** Bar graph showing average changes in *I*_*sc*_ responding to kaempferol alone and after pretreatment with BAPTA-AM. **(C)** Representative tracings of apical *I*_*Cl*_ induced by kaempferol (50 μM, apical and basolateral) after bilateral pretreatment with protein kinase A inhibitor H89 (10 µM), tyrosine kinase inhibitors AG490 (10 µM) or tyrphostin A23 (100 µM) and tyrosine phosphatase vanadate (100 µM). **(D)** Bar graph showing average changes in apical *I*_*Cl*_ responding to kaempferol alone and after pretreatment with these inhibitors. Values are expressed as mean ± SD (n = 4). *P < 0.05 compared with kaempferol alone by one-way ANOVA and Dunnett’s *post-hoc* test.

To determine whether the Cl^−^ secretion induced by kaempferol was dependent of intracellular Ca^2+^, the kaempferol response was examined in the presence of BAPTA-AM, a membrane-permeable Ca^2+^ chelator. Pretreatment with BAPTA-AM (50 μM, apical and basolateral) for 30 min did not change the baseline. A subsequent addition of kaempferol increased the *I*_*sc*_ response to 18.05 ± 2.25 μA/cm^2^ (n = 4, Figures 11A,B). The kaempferol-activated *I*_*sc*_ in the presence of BAPTA-AM was not significantly different compared with kaempferol alone (21.55 ± 5.82 μA/cm^2^; Figure 11B). This evidence suggests that the effect of kaempferol on activating Cl^−^ secretion may not involve the Ca^2+^ mobilization but possibly relate to the cAMP signaling pathway.

### 3.7 Cellular mechanism of kaempferol mediating Cl^−^ secretion

Cl^−^ secretion in colonic epithelium occurring mainly through CFTR has been reported to be regulated by PKA or protein tyrosine kinase (PTK) phosphorylation and protein tyrosine phosphatase (PTT) dephosphorylation. To further investigate the mechanisms of kaempferol on regulating Cl^−^ secretion via protein kinases and phosphatases, the kaempferol-activated apical *I*_*Cl*_ was determined in the presence of kinase or phosphatase inhibitors for 30 min in apical and basolateral solutions as shown in Figure 11C. Pretreatment with H89 (10 µM), a PKA inhibitor, significantly decreased the kaempferol-activated *I*_*Cl*_ by 88% (−7.15 ± 4.25 μA/cm^2^, n = 4, *P* < 0.05, Figures 11C,D). AG490 (10 µM) or tyrphostin A23 (100 µM), PTK inhibitors, inhibited the kaempferol-activated *I*_*Cl*_ by 16% (−50.04 ± 6.03 μA/cm^2^, n = 4) or by 16% (−50.00 ± 1.32 μA/cm^2^, n = 4), respectively. Vanadate (100 µM), a PTT inhibitor, increased the kaempferol-activated *I*_*Cl*_ by 3% (−61.66 ± 7.16 μA/cm^2^, n = 4). Nonetheless, the kaempferol response in the presence or absence of AG490, tyrphostin A23, or vanadate was not statistically significant (Figure 11D). Our results reveal the mechanism of kaempferol action via a protein kinase A activity.

### 3.8 Effect of kaempferol on CFTR protein expression

**FIGURE 12:**
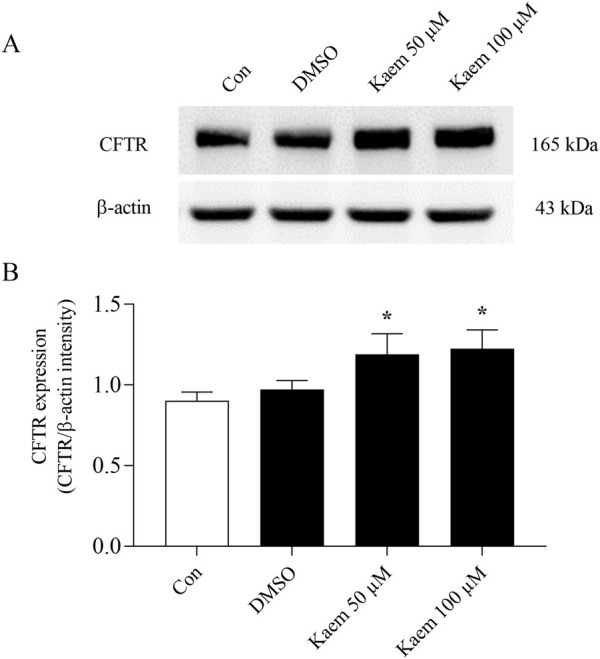
Effect of kaempferol on the expression of CFTR protein in T84 cells. Total proteins were isolated from the cells treated with DMSO or kaempferol at 50 and 100 µM for 24 h to examine the CFTR protein expression by Western blot analysis. **(A)** Representative Western blot images of CFTR and β-actin protein expression. **(B)** Quantification of CFTR expression using densitometric analysis was normalized to housekeeping protein β-actin. Values are expressed as mean ± SD (n = 5). *P < 0.05 compared with DMSO by one-way ANOVA and Dunnett’s *post-hoc* test.

To examine the effect of kaempferol on regulating Cl^−^ transport protein, the expression of CFTR was analyzed by Western blot analysis. T84 cells were cultured in standard media for 7 days before treatment with kaempferol (50 or 100 µM) or DMSO for 24 h. The protein bands demonstrated CFTR and β-actin with a molecular weight of 165 and 43 kDa, respectively (Figure 12A). The protein band density measured as the expression ratio of CFTR to β-actin revealed a slight but not statistically significant increase in the CFTR expression in the DMSO group compared with the control group that received no treatment (Figure 12B). Kaempferol 50 and 100 µM treatment significantly increased CFTR expression by 23% (1.20 ± 0.13, n = 5, *P* < 0.05, Figure 12B) and 25% (1.22 ± 0.12, n = 5, *P* < 0.05, Figure 12B) compared with DMSO, respectively. The kaempferol effect upregulates CFTR expression according to the increased concentration.

## 4 Discussion

The major finding of our study using T84 as a cell model showed that kaempferol stimulates Cl^−^ secretion occurred primarily via CFTR with no effect on electrogenic Na^+^ absorption. The mechanisms of kaempferol appeared to modulate the phosphorylation of PKA and upregulation of CFTR expression.

The effect of kaempferol on stimulation of Cl^−^ secretion in T84 cells was supported by experiments in both intact and permeabilized monolayers. Firstly, in intact monolayer, kaempferol-activated *I*_*sc*_ was mostly inhibited by commonly used CFTR inhibitors, NPPB and glibenclamide (Figures 3C,D). Notably, pretreatment with a selective CFTR inhibitor, CFTRinh-172, failed to inhibit the kaempferol-stimulated *I*_*sc*_ while posttreatment reduced the maximum kaempferol response by 53% (Figure 3A). This result shows that a typical *I*_*sc*_ response to kaempferol can be obtained with little active CFTR following pretreatment with CFTRinh-172. Alternatively, kaempferol may stimulate other anion channels or pathways that could compensate for inactive CFTR. Similarly, activation of *I*_*Cl*_ by kaempferol in the permeabilized monolayer with high Cl^−^ gradient was inhibited completely by CFTRinh-172 and partially by CaCC inhibitors, CaCCinh-A01 (Figures 8B,C). Secondly, kaempferol induced increases in *I*_*sc*_ and *I*_*Cl*_ were insensitive to the Na^+^ channel blocker, amiloride (Figures 3A, 8A). Thirdly, anion substitution experiments verified Cl^−^ dependency of kaempferol effect (Figures 6A,B). Fourthly, a significant reduction of the kaempferol-activated *I*_*sc*_, by the NKCC inhibitor bumetanide (Figure 4C) which was known to inhibit Cl^−^ entry across the basolateral membrane indicates a role of NKCC in mediating kaempferol-induced Cl^−^ secretion. Lastly, kaempferol increased *I*_*Kb*_, which was substantially inhibited by BaCl_2_ and clotrimazole, a non-selective inhibitor of K^+^ channels and Ca^2+^-activated K^+^ channels. Activation of basolateral K^+^ current helps to maintain an electrochemical driving force, which is required for NKCC function to increase Cl^−^ secretion. All these observations were consistent with the activation of electrogenic Cl^−^ secretion probably through CFTR in T84 cells (Nguyen et al., 1991). In addition, several flavonoids have been demonstrated to increase Cl^−^ secretion via activation of the basolateral K^+^ channel in numerous cell types (Deachapunya and Poonyachoti, 2013; Hao et al., 2015; Yu et al., 2016).

Activation of Cl^−^ secretion by kaempferol occurred predominantly via CFTR channels. This was evidenced by our findings that the kaempferol-stimulated *I*_*sc*_, was mostly inhibited by posttreatment with CFTR blockers and less inhibited by CaCC blockers, and that the kaempferol-activated apical *I*_*Cl*_ was completely inhibited by both pretreatment and posttreatment with CFTRinh-172. However, a substantial decrease in the kaempferol-induced *I*_*Cl*_ before and after CaCCinh-A01 indicates some contribution of CaCC in the kaempferol response. Since the kaempferol induced increase in the *I*_*sc*_ did not involve Ca^2+^ mobilization, it raises the possibility that kaempferol may at least modulate protein kinases required for CaCC-mediated Cl^−^ current, which remains to be studied. All these results along with the finding that the kaempferol-stimulated *I*_*Cl*_ was mostly inhibited by H89 provide reasonable evidence to support kaempferol stimulation of Cl^−^ secretion mainly through CFTR and, to a less extent, CaCC.

Kaempferol was found to modulate cAMP-activated Cl^−^ secretion. The increase in *I*_*sc*_ induced by forskolin was markedly inhibited by a subsequent addition of kaempferol (Figure 7A) which seems to contradict its stimulatory effect on basal *I*_*sc*_ (Figure 2C). Although the inhibitory effect of kaempferol corresponded to studies with quercetin, morin, and naringenin that have been shown to inhibit the forskolin-stimulated *I*_*sc*_ in multiple cell types (Schuier et al., 2005), the underlying mechanism is unknown. Forskolin directly activates adenylate cyclase to generate cAMP levels and activate PKA. The potent activation may saturate the signaling pathways, thereby diminishing the subsequent effects of other drugs that rely on cAMP and PKA mechanisms (Levijoki et al., 2022).

Nevertheless, kaempferol had no additive effect on cAMP-stimulated apical *I*_*Cl*_ induced by forskolin or cAMP-analog 8cpt-cAMP (Figures 10A,B). Relating the explanation of genistein and quercetin effects in T84 and CFBE41o^−^ cells (Illek et al., 1996; Pyle et al., 2010), non-additive effects of kaempferol and cAMP agonists may be at least an influence on a common Cl^−^ secretory pathway involving cAMP-dependent Cl^−^ secretion via CFTR. Due to cellular cAMP not being measured, it could be possible that kaempferol and cAMP agonists may share an activation of PKA activity in mediating Cl secretion through CFTR.

Although stimulation of Cl^−^ secretion through CaCC was less influenced by kaempferol, kaempferol appears to modulate Ca^2+^-activated Cl^−^ secretion induced by carbachol (CCh), a muscarinic agonist known to activate Cl^−^ secretion through CaCC. CCh produced a slight increase in *I*_*sc*_ under basal condition but markedly increased after pretreatment with kaempferol (Figure 7B). Conversely, the kaempferol effect was potentiated after CCh (Figure 7C). This additive effect of kaempferol and CCh suggests that kaempferol is unlikely to increase Cl^−^ secretion via an increase in intracellular Ca^2+^. In agreement with our study, CCh at 1–100 μM alone was previously shown to provoke a minor stimulation of *I*_*sc*_, but potentiate the *I*_*sc*_ changes in cells that had been pre-stimulated with adenosine 3′,5′-cyclic monophosphate (cAMP) agonists, such as prostaglandins (Warhurst et al., 1991). Although the mechanism is unknown, it could be possible that amplifying the CCh-stimulated *I*_*sc*_ after kaempferol is due to priming effect of kaempferol on cAMP/PKA. The reverse order may be the prime effects of CCh-induced Ca^2+^ and PKC activation.

In the present study, a PKA inhibitor H89 completely inhibited the kaempferol-increased *I*_*Cl*_ (Figure 11C), indicating that the stimulatory effect of kaempferol on Cl^−^ secretion requires PKA-dependent phosphorylation of CFTR. Consistently, inhibition of PKA activity by H89 has been shown to reduce Cl^−^ secretion in T84 cells (Hoque et al., 2010). However, tyrosine kinase inhibitors tyrphostin A23 and AG490 and a tyrosine phosphatase inhibitor vanadate did not affect the increase in *I*_*Cl*_ induced by kaempferol, suggesting that kaempferol-induced Cl^−^ secretion is not mediated by tyrosine phosphorylation or dephosphorylation.

Our study revealed that treatment of kaempferol (50 and 100 µM) for 24 h upregulated CFTR protein expression in T84 cells (Figure 12). Although the molecular mechanism of kaempferol action on CFTR remains to be elucidated, it is possible that various processes, including transcriptional regulation, enhanced translation efficiency, or reduced protein degradation could be the targets of phytochemical on CFTR expression (Baharara et al., 2023). A previous study in BHK cells demonstrated the genomic effect of isoflavone genistein on CFTR mediated by estrogen receptors (Schmidt et al., 2008). In addition, cAMP-PKA pathways significantly involve posttranslational increases in CFTR expression (Liang et al., 2012). It raises the possibility that phytoestrogen kaempferol may upregulate CFTR expression via the estrogen receptor-mediated pathway. Alternatively, the proposed cAMP/PKA signaling pathway in mediating the kaempferol-induced CFTR activity may also be associated with the increased CFTR expression by kaempferol.

Dietary kaempferol is usually present in glycosidic forms with different polarities. Glucosides with low-polarity, but not high-polarity glycosides, are easily absorbed. The kaempferol content in foods vary significantly from 47 to 234 mg per 100 g depending on the type of food and its preparation (Miean and Mohamed, 2001; Zhou et al., 2011). Kaempferol which is more stable under acidic than alkaline conditions (Qiu et al., 2020) can be absorbed primarily in the small intestine via passive diffusion, facilitated diffusion (Alam et al., 2020), or could be absorbed through transporters of the human organic anion transporting polypeptide (OATP) located in the apical membrane of human enterocytes (Mandery et al., 2010). However, the maximum plasma concentration of kaempferol has been revealed at 0.1 µM after oral administration of 9 mg (DuPont et al., 2004). High concentrations of flavonoids found in the intestinal lumen also suggest that they are poorly absorbed in the human digestive tract (Sun et al., 2014). Intestinal enzymes metabolize kaempferol in the small intestine to kaempferol-glucuronide. A study on the metabolism of flavonoids, including kaempferol, revealed that a fraction of kaempferol remains unabsorbed and passes into the colon where a substantial metabolic transformation occurs; as a result, a small fraction of flavonoids reaches the colon as compared to its initial intake (Serra et al., 2012).

Our finding showed that kaempferol produced Cl^−^ secretion when applied to the apical (luminal) side to a greater extent than to the basolateral (blood) side of the colonic monolayer. The evidence suggests that the luminal concentration of kaempferol reaching the colon should be considered rather than the kaempferol concentration in the plasma. Therefore, the effective concentration as low as 5 μM retrieved from the present *in vitro* study could predict the therapeutic effect of kaempferol-induced chloride secretion in humans. It is noted that the concentration of kaempferol in the human colon is significantly lower than those currently used in our study, likely due to the microbial digestive process by the commensal bacteria. Oral supplementation of kaempferol to activate Cl^−^ secretion *in vivo* study should be further studied.

**FIGURE 13:**
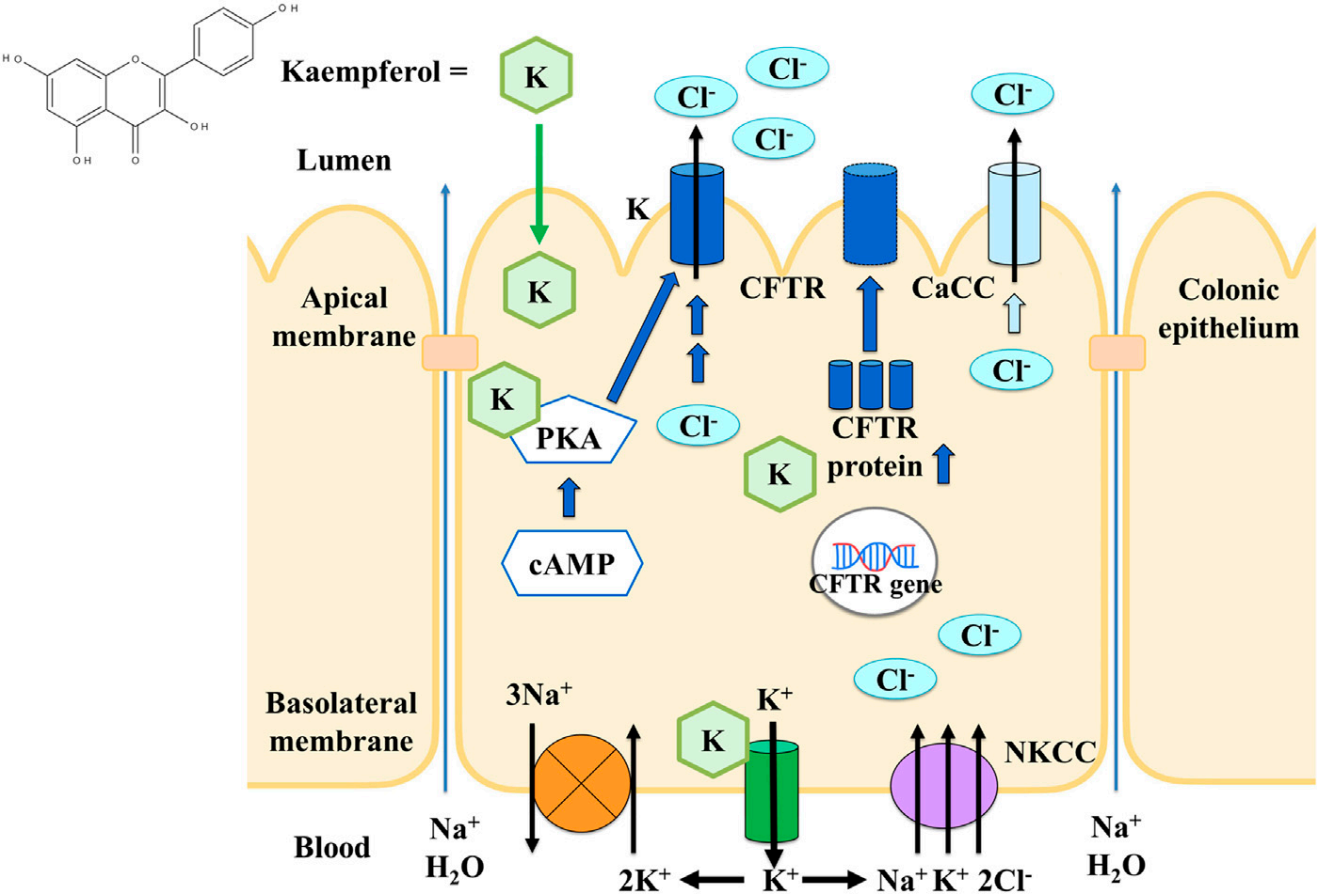
The model of the effect and cellular mechanism of kaempferol on activating Cl^−^ secretion in colonic epithelium.

Overall, the model of Cl^−^ secretion activated by kaempferol in the colonic epithelium was summarized in Figure 13. Kaempferol is absorbed via the apical membrane to increase apical Cl^−^ current mainly through CFTR and partially through CaCC allowing Cl^−^ exit the cells. Kaempferol also activates basolateral K^+^ current via K^+^ channels which provides hyperpolarizing driving force for apical Cl^−^ exit. Activity of NKCC is also required for kaempferol. The basolateral K^+^ exit works together with NKCC and Na^+^-K^+^-ATPase to produce an electrochemical driving force for Cl^−^ secretion. The mechanism of kaempferol on stimulating Cl^−^ secretion appears to involve the activation of PKA which phosphorylates and opens CFTR as well as increase CFTR expression. The increased Cl^−^ secretion by kaempferol will promote Na^+^ and water into the colonic lumen through the paracellular pathway which could be useful for increasing stool fluidity and relieving constipation.

## 5 Conclusion

Kaempferol activates Cl^−^ secretion in T84 cells by stimulating apical Cl^−^ current predominantly via CFTR and basolateral K^+^ current. The kaempferol-stimulated Cl^−^ secretion seems to involve cAMP/PKA signaling pathway and increase CFTR expression. Our findings provide functional information of the effect and cellular mechanisms of kaempferol on regulating Cl^−^ secretion and thereby fluid secretion in the colonic epithelial cells. The beneficial effects of kaempferol could be pharmaco-therapeutically applied for the treatment of constipation.

## Data Availability

The raw data supporting the conclusions of this article will be made available by the authors upon request, without undue reservation.
